# The Risk for Schizophrenia–Bipolar Spectrum: Does the Apple Fall Close to the Tree? A Narrative Review

**DOI:** 10.3390/ijerph20156540

**Published:** 2023-08-07

**Authors:** Giulia Cattarinussi, Alessio A. Gugliotta, Fabio Sambataro

**Affiliations:** 1 Department of Neuroscience (DNS), University of Padova, 35131 Padova, Italy; giulia.cattarinussi@unipd.it (G.C.); alessio.gugliotta@gmail.com (A.A.G.); 2 Padova Neuroscience Center, University of Padova, 35131 Padova, Italy

**Keywords:** psychosis, high risk, relatives, cognitive functions, neuroimaging, fMRI, MRI

## Abstract

Schizophrenia (SCZ) and bipolar disorder (BD) are severe psychiatric disorders that share clinical features and several risk genes. Important information about their genetic underpinnings arises from intermediate phenotypes (IPs), quantifiable biological traits that are more prevalent in unaffected relatives (RELs) of patients compared to the general population and co-segregate with the disorders. Within IPs, neuropsychological functions and neuroimaging measures have the potential to provide useful insight into the pathophysiology of SCZ and BD. In this context, the present narrative review provides a comprehensive overview of the available evidence on deficits in neuropsychological functions and neuroimaging alterations in unaffected relatives of SCZ (SCZ-RELs) and BD (BD-RELs). Overall, deficits in cognitive functions including intelligence, memory, attention, executive functions, and social cognition could be considered IPs for SCZ. Although the picture for cognitive alterations in BD-RELs is less defined, BD-RELs seem to present worse performances compared to controls in executive functioning, including adaptable thinking, planning, self-monitoring, self-control, and working memory. Among neuroimaging markers, SCZ-RELs appear to be characterized by structural and functional alterations in the cortico–striatal–thalamic network, while BD risk seems to be associated with abnormalities in the prefrontal, temporal, thalamic, and limbic regions. In conclusion, SCZ-RELs and BD-RELs present a pattern of cognitive and neuroimaging alterations that lie between patients and healthy individuals. Similar abnormalities in SCZ-RELs and BD-RELs may be the phenotypic expression of the shared genetic mechanisms underlying both disorders, while the specificities in neuropsychological and neuroimaging profiles may be associated with the differential symptom expression in the two disorders.

## 1. Introduction

In 1899, the German psychiatrist Emil Kraepelin postulated the dichotomy between “dementia praecox” and “manic depressive insanity”, paving the way for the psychiatric diagnoses of the twentieth century [1]. Later in his life, Kraepelin himself admitted that this clear distinction did not apply to a large number of patients who presented features of both of these disorders [2]. In 1933, the term “schizoaffective psychosis” was first used to describe a disorder characterized by psychotic and affective features [3]. In the following decades, alternative diagnostic frameworks were proposed, including the idea of psychosis as a continuum that extends from unipolar through bipolar disorders (BD) and schizoaffective disorder (SZA) to schizophrenia (SCZ) [4]. This theory, first proposed in 1986, is supported by recent genetic research that has shown that SCZ and BD, which have a high genetic component, also share risk genes [5]. In light of this evidence, the study of intermediate phenotypes (IPs) has proven to be particularly informative, as they provide an index of liability that is more amenable to study than the disorders themselves [6]. In particular, IPs are heritable biological traits underlying complex phenotypes that co-segregate with the disorder: they are present in unaffected relatives (RELs) at a higher rate than in the general population and are state-independent [6].

Within candidate IPs, cognitive functioning and neuroimaging measures have shown promising results for SCZ and BD. Regarding cognitive abilities, a large body of research has reported cognitive impairments in SCZ and BD in a broad range of domains, including the intelligence quotient (IQ), executive functions, memory, and attention. In particular, meta-analytic evidence has shown that SCZ patients perform worse than healthy controls (HC) in general intelligence, verbal and non-verbal memory, visual and auditory attention, spatial ability, executive functions, and language [7]. More recently, a meta-analysis including 18,300 cases detected cognitive deficits in SCZ compared to HCs in global cognitive functioning, memory, language, executive functions, and attention [8]. The picture in BD is less univocal: numerous studies have shown that some individuals perform worse compared to HC, while others appear to outperform the unaffected population [9]. Interestingly, a history of childhood maltreatment, early onset of the disorder, psychotic and residual depressive symptoms, and a higher number of previous manic episodes appear to significantly affect cognitive performance in patients with BD [9,10,11]. Studies that have compared cognitive functioning in SCZ and BD have demonstrated that patients with SCZ present more severe cognitive dysfunctions, particularly in attention and social cognition [12]. Remarkably, these differences have also been observed in the early phases of the disorders, with patients with first-episode SCZ (FES) significantly underperforming relative to patients with first-episode BD (FEBD) in most cognitive domains. Interestingly, this meta-analysis also showed that neuropsychological impairments of FEBD, which were comparable to chronic BD, are intermediate between FES and HCs [13].

In addition to cognitive alterations, in the last decades, the study of neuroimaging abnormalities in SCZ and BD has gained increasing popularity, and now a large body of literature on this topic is available. In SCZ, structural and functional alterations in the regions of the fronto-temporal [14] and fronto-parietal executive network have been commonly reported, and they appear to be predictive of executive function performances [15]. A recent meta-analysis has highlighted abnormalities in SCZ in several other brain networks, including the default mode network (DMN), salience, subcortical, visual, sensorimotor, and emotional networks, and these alterations may represent the neurobiological underpinnings of cognitive deficits [16]. In BD, reductions in grey matter volume have been reported in the right ventral prefrontal cortex, insula, temporal cortex, and claustrum [17], in addition to hypoactivation of the inferior frontal cortex and putamen and overactivation of limbic areas, including the parahippocampal gyrus, hippocampus, and amygdala, and basal ganglia [18]. Similar to chronic patients, first-episode psychosis (FEP) is associated with alterations in the thalamocortical network, default mode, salience, and executive networks [19,20]; nonetheless, these cortical changes show a progression from FEP through chronic SCZ [21].

Unfortunately, both cognitive functioning and neuroimaging measures in patients are affected by several confounding factors. For instance, there is ample evidence that pharmacological treatments influence gray matter and white matter parameters, as well as brain activation and connectivity [22,23,24]. Furthermore, the use of medications seems to be also associated with changes in cognitive abilities [25,26]. In addition, lifestyle factors, including sedentary lifestyle, smoking, and substance use, appear to be implicated in cognitive impairments in patients with SCZ and BD [27,28,29] and severely affect brain structure and function [30,31]. In this context, the study of IPs in unaffected relatives may help to clarify the pathophysiology of these disorders without the presence of confounding effects related to the disorder.

The overarching goal of the present narrative review is to provide a comprehensive and updated overview of the available evidence on cognitive deficits and neuroimaging alterations in unaffected relatives of SCZ (SCZ-RELs) and BD (BD-RELs). We aim to expand on the results of the existing literature in three important ways: (a) to report common cognitive changes and brain abnormalities evaluated with structural magnetic resonance imaging (MRI) and functional MRI (fMRI) in SCZ-RELs and BD-RELs compared to HCs, highlighting their role as potential markers of risk or resilience for the two disorders; (b) to identify disorder-specific cognitive abnormalities and brain structural and functional alterations in SCZ-RELs compared to BD-RELs, in order to clarify the specific pathophysiological paths that are associated with the risk for SCZ and BD, respectively; and (c) to analyze current literature with a transdiagnostic approach, by discussing studies assessing relatives of patients with affective and non-affective psychosis (PSY-RELs).

In the following sections, we first present evidence for SCZ-RELs and BD-RELs separately, then we discuss studies assessing PSY-RELs, including studies directly comparing SCZ-RELs and BD-RELs. The main neuropsychological and neuroimaging findings in SCZ-RELs and BD-RELs can be found in Table 1.

## 2. Methods

### 2.1. Search Strategy

A search of the literature on PubMed and Web of Science was conducted in April 2023 without language restrictions. The following keywords were employed: (“schizophrenia”[Title/Abstract] OR “bipolar disorder”[Title/Abstract]) AND (“relatives”[Title/Abstract] OR “first degree relatives”[Title/Abstract] OR “genetic risk”[Title/Abstract] OR “twins”[Title/Abstract] OR “offspring”[Title/Abstract] OR “parents”[Title/Abstract] OR “liability”[Title/Abstract] OR “family study”[Title/Abstract])) AND (“neuroimaging”[Title/Abstract] OR “brain imaging”[Title/Abstract] OR MRI[Title/Abstract] OR fMRI[Title/Abstract] OR “magnetic resonance”[Title/Abstract] OR “cognition”[Title/Abstract] OR “cognitive”[Title/Abstract] OR “neuropsychology”[Title/Abstract] OR “intelligence”[Title/Abstract] OR “IQ”[Title/Abstract] OR “memory”[Title/Abstract] OR “attention”[Title/Abstract] OR “executive function”[Title/Abstract] OR “working memory”[Title/Abstract] OR “social cognition”[Title/Abstract]). We also included relevant studies appearing in the reference lists of the selected articles. 

### 2.2. Inclusion and Exclusion Criteria

Studies were included if they met the following inclusion criteria: (a) original peer-reviewed articles; (b) they included a group of unaffected RELs of patients with SCZ, BD, or PSY; (c) they explored cognitive functioning or structural and/or functional MRI alterations. We excluded: (a) studies exploring brain metabolism, perfusion, or electrophysiology; (b) studies conducted on animals. As a narrative review should allow for the broadest possible approach, we did not apply additional exclusion criteria, but instead we evaluated articles regarding their scientific merit and relevance. Reviews, meta-analyses, and mega-analyses were included, since they provide an updated qualitative and quantitative synthesis of the current knowledge. Once we had identified the main cognitive domains and neuroimaging alterations associated with the risk for SCZ and BD, we conducted additional literature searches in PubMed to specifically investigate these topics. Consequently, this narrative review provides qualitative rather than quantitative insights into endophenotypes for SCZ and BD.

## 3. Neuropsychological Findings in SCZ-RELs

### 3.1. Intelligence

The intellectual impairment observed in SCZ appears to be linked to the genetic risk for the disorder, and it is commonly considered an IP. Indeed, a large body of the literature has highlighted that SCZ-RELs have a lower IQ compared to the general population [32,33,34,35]. In addition to intellectual impairment, the superiority of verbal skills over spatial skills has been reported in this population [36]. A meta-analysis of 28 studies on young first-degree SCZ-RELs who had not reached the peak age illness risk (<age 30 years) demonstrated that SCZ-RELs presented deficits with a moderate level of severity compared with HC, with the largest average effect sizes for full-scale IQ, vocabulary, and single-word reading tests (often used as estimates of IQ) [37]. In addition to a lower full-scale IQ, young SCZ-RELs also exhibited significantly poorer scholastic achievement compared to typically developing children [38]. Interestingly, in a study that examined the association between a family history of psychosis, depression, mania, and alcohol or substance abuse and IQ in SCZ, siblings of SCZ and HCs reported a decrease in IQ in the sibling group compared to HCs, which was not influenced by family history [39]. Notably, lower IQ in SCZ-RELs seems to be associated with microstructural changes in sleep architecture, primarily sleep spindle deficits, that are, therefore, considered IPs contributing to cognitive dysfunction in SCZ [40,41].

A deeper understanding of the intellectual impairment of SCZ-RELs is derived from twin studies. In particular, twin studies allow us to assess genetic versus non-genetic components, heritability, and shared genetic variances in cognitive performance [42]. A study exploring intelligence in 267 monozygotic (MZ) and dizygotic (DZ) twins concordant and discordant for SCZ and healthy MZ and DZ control twin pairs demonstrated a substantial genetic contribution to intelligence [43]. Interestingly, a longitudinal study with a 5-year follow-up period conducted in twins discordant for SCZ showed a stable course in IQ over time in SCZ relative to both the unaffected co-twins and healthy control twins, who showed a small increase. The change in IQ change in the unaffected co-twins was comparable to that in healthy control twins [44]. Moreover, it has been shown that, in MZ and DZ twin pairs with SCZ, divergence in school performance between the affected and unaffected twin pairs occurred at 12 years of age, approximately 7.5 years earlier than it did in control twins, preceding the onset of psychosis by 10 years. As expected, in the affected twin pairs, developmental divergence occurred mainly due to the underperformance of the SCZ twin [45].

An investigation that explored the relationship between structural brain abnormalities and IQ in five SCZ family cohorts observed that offspring, siblings, and MZ co-twins had a significantly lower IQ than HCs, while the MZ co-twins had a significantly higher IQ. First-degree SCZ-RELs presented smaller intracranial volume (ICV), surface area (SA), total brain, gray matter, cerebral white matter, cerebellar gray and white matter, thalamus, putamen, amygdala, and accumbens volumes compared to HC. Notably, these changes strongly covaried with IQ, suggesting that the observed brain alterations in SCZ-RELs were at least partly explained by genes associated with both SCZ risk and IQ [46]. Similarly, a study carried out on a smaller sample showed that, in non-psychotic SCZ-RELs, there was an association between left hippocampal volume and performance IQ [47].

### 3.2. Memory, Attention, and Executive Functions

Overall, the available evidence seems to univocally suggest that cognitive performances in SCZ-RELs lie intermediately between SCZ and HCs. A meta-analysis conducted on 2872 SCZ-RELs and 2457 HCs showed cognitive deficits in SCZ-RELs in a variety of tasks, with the largest effect sizes observed in continuous performance tasks, auditory verbal learning, design copy tests, and category fluency. Additionally, SCZ-RELs presented worse performances in tasks with high executive control demands, such as working memory, set-shifting, and response inhibition [48]. Another meta-analysis explored the executive functions of SCZ-RELs by collecting available evidence on studies using the Wisconsin Card Sorting Test, the Trail Making Test, the Stroop Test, and the Verbal Fluency Test. Results showed worse performances of SCZ-RELs on all executive tests, with greater effect estimates for the fluency tests [49]. Moreover, studies exploring memory with the Wechsler Memory Scale and the California Verbal Learning Test demonstrated that SCZ-RELs presented deficits in the verbal paired associates test, the logical stories test, the digit span forward and backward tests, while no differences were reported in tasks of delayed recall [50].

Interestingly, a recent investigation on the influence of copy number genetic variants (CNVs) on cognitive IPs for SCZ displayed that carriers of specific schizophrenia-associated CNVs showed poorer performance than non-carriers in immediate and delayed verbal recall and block design performance [51]. Moreover, higher polygenic risk score (PRS) for SCZ indicating genetic liability to the disorder seems to be associated with poorer cognitive abilities evaluated with the MATRICS Consensus Cognitive Battery [52].

In line with studies conducted on siblings, offspring, and parents of SCZ, investigations on twins discordant for the disorder have highlighted the presence of widespread cognitive deficits in subjects at genetic risk for SCZ. In particular, a twin study that examined the heritability of working memory, processing speed, perceptual organization, and verbal comprehension indicated that genetic influences contributed substantially to all cognitive domains, but working memory was the most heritable [43]. Similarly, a study on 418 MZ and DZ twins, including pairs concordant and discordant for SCZ, demonstrated that genetic influences contributed substantially to mental flexibility [53]. Notably, a recent meta-analysis of 170 published twin and family studies demonstrated that heritability ranged across cognitive domains, likely due to differences in genetic and environmental effects, with the highest heritability for general cognitive ability, verbal ability, visuospatial ability, attention, and speed of processing, while the lowest heritability was observed for executive functions. In addition, the results also showed that the heritability of cognitive phenotypes did not differ between SCZ and nonpsychiatric populations, suggesting that SCZ samples could be considered valuable for studying the genetic basis of cognitive impairment in patients [54]. As demonstrated by Toulopoulou and colleagues, genes associated with SCZ considerably overlap with genes involved in cognition [55]. Moreover, the same research group found that cognitive deficits lay upstream of the liability for SCZ, with about 25% of the variance in liability to SCZ explained by variation in cognitive function [56]. Consistently with these results, a recent nation-wide twin study showed significant heritability for planning/spatial span, spatial working memory, sustained attention, and movement time, while only environmental factors contributed to set-shifting, reflection impulsivity, and thinking time. Liability to SCZ was associated with planning/spatial span, spatial working memory, sustained attention, and set-shifting [57].

Interestingly, an investigation exploring sex differences in cognition among SCZ-RELs showed that women outperformed men in immediate verbal reproduction and in the use of semantic clustering as a learning strategy. When SCZ patients were included in the analyses, women performed better than men in processing speed, set-shifting, and verbal episodic memory, whereas men outperformed women in visual working memory [58].

### 3.3. Social Cognition

Studies exploring social cognition and its sub-domains (mentalizing, emotional processing, social perception, social knowledge, and attributional style) have demonstrated global deficits in SCZ-RELs [59]. Furthermore, two different meta-analyses have reported modest to moderate effects of theory of mind (ToM) impairments in unaffected SCZ-RELs [60,61]. A meta-analysis of 29 studies on adult first-degree SCZ-RELs confirmed a general deficit in social cognition, particularly in mentalizing and emotional processing and perception [62]. In addition, non-psychotic first-degree SCZ-RELs presented significantly lower emotional intelligence compared to HCs [63].

Evidence from twin studies reports similar results, showing alterations in social cognition, in particular in the ability to detect sarcasm, in unaffected MZ co-twins, indicating that social cognitive deficits could be a genetic vulnerability indicator of the disease [64].

## 4. Neuropsychological Findings in BD-RELs

### 4.1. Intelligence

A narrative review of 18 publications did not show consistent evidence of a lower or higher IQ in the offspring of parents diagnosed with BD, with some tentative evidence for a lower IQ performance in the offspring of BD compared to HCs [65]. A recent study showed that siblings of BD presented lower IQ compared to HCs but similar educational performance [66]. Overall, intelligence in BD-RELs does not seem to represent an IP for the disorder.

### 4.2. Memory, Attention, and Executive Functions

Previous publications have reported cognitive deficits in BD-RELs across executive functioning, including proficiency in adaptable thinking, planning, self-monitoring, self-control, and working memory [67,68]. Similarly, another meta-analysis showed that first-degree BD-RELs had worse executive functions and verbal memory compared to HC, although the effect size was small [69]. The offspring of BD parents display greater deficits in memory, cognitive flexibility, and social cognition compared to the offspring of unaffected individuals [70]. Interestingly, Bora and colleagues observed that BD-RELs had significant impairment in verbal working memory and executive functions, while verbal memory and psychomotor performance did not differ from HC. In addition, they reported that one component of executive functions, cognitive flexibility, was associated with a family history of BD with psychotic features [71]. A meta-analysis conducted by the same research group indicated that deficits in response inhibition, set-shifting, executive function, verbal memory, and sustained attention were common features for both BD and BD-RELs, although with a small effect size. Differently, processing speed, visual memory, and verbal fluency deficits were only observed in BD [72]. Crucially, a recent systematic review exploring the association between cognitive performances and subsequent illness onset reported that in first-degree BD-RELs, impairments in attention, verbal memory, and executive functions and positive bias were predictive of the development of manic, hypomanic, or depressive episodes [68].

### 4.3. Social Cognition

A meta-analysis of ToM and facial emotion recognition in BD-RELs, including 728 first-degree BD-RELs and 865 HCs, showed a significant impairment of ToM in BD-RELs, although the effect size for the difference between BD-REL and HCs was small [73]. More evidence is needed to conclude that social cognitive abilities represent an IP of BD.

## 5. Neuropsychological Findings in PSY-RELs

### 5.1. Intelligence

A meta-analysis exploring cognition in young PSY-RELs, individuals at ultra-high risk for psychosis (UHR), and HCs highlighted that subjects at genetic and clinical risk for psychosis had lower IQ compared to HCs. In both risk groups, the presence of attenuated psychotic symptoms was associated with more severe cognitive impairments [74]. 

The Enhancing Neuro Imaging Genetics Through Meta-Analysis (ENIGMA)-Relatives Working Group performed a meta-analysis of cognitive and MRI data of SCZ, BD, first-degree SCZ-RELs, first-degree BD-RELs, and HCs. SCZ-RELs showed a similar pattern of reduced IQ to SCZ, albeit with smaller effect sizes. BD-RELs showed mild IQ reductions compared to HCs with a borderline significance level. Interestingly, both REL groups had similar educational attainment compared to HCs [75]. Premorbid IQ was also tested in a study on SCZ, AFF-PSY (BD and SZA), first-degree SCZ-RELs, first-degree AFF-PSY-RELs, and HC. Overall, SCZ showed a lower premorbid IQ than SCZ-RELs, AFF-PSY, and HCs; BD had a lower premorbid IQ than AFF-PSY-RELs, but SZA patients did not, and neither group differed significantly from HCs. The RELs of schizophrenic patients who had obstetric complications had a significantly higher IQ compared to relatives of schizophrenic patients without obstetric complications [76].

### 5.2. Memory, Attention, and Executive Functions

Studies in individuals at genetic risk for psychosis have consistently described verbal memory impairments in PSY-RELs [77,78,79]. In particular, verbal memory deficits were reported in the context of a larger cognitive impairment, including impairments in the domains of attention [77,78,79,80] and executive function and working memory [77,78,79,81]. Notably, a study comparing PSY-RELs with UHR, FEP, and HCs showed that functioning in verbal memory, attention, processing speed, and working memory displayed a gradual decrease from HCs, to PSY-RELs, UHR and FEP, showing that cognitive functioning in the PSY-RELs group was intermediate between HCs and UHR [78]. Deficits in verbal memory were also reported in young PSY-RELs and UHR, along with poorer visual memory, executive functions, fluency, sustained attention, and verbal and visuospatial working memory. Interestingly, these differences were even more pronounced in the presence of attenuated psychotic symptoms [74]. Furthermore, evidence from longitudinal studies shows that a greater impairment of verbal memory in PSY-REL at baseline was associated with the development of psychotic symptoms at follow-up, while IQ and executive functions did not predict the onset of psychosis [82]. 

In addition to verbal memory impairments, alterations in working memory have been commonly reported, both in young PSY-RELs [83] and in adult PSY-RELs [84]. In particular, in a sample of 289 SCZ, 227 psychotic BD, 165 SZA, their first-degree RELs (*n* = 315, *n* = 259, *n* = 193, respectively), and 289 HCs from the Bipolar and Schizophrenia Network on Intermediate Phenotypes study, patients, and RELs showed a similar pattern of working memory deficits [84]. A study comparing young unmedicated first-degree SCZ-RELs and RELs of patients with affective psychosis (AFF-PSY-RELs) demonstrated that, compared to HCs, SCZ-RELs had worse working memory performances, whereas AFF-PSY-RELs presented impairments in auditory vigilance. Adjusting for physical anhedonia, phobic anxiety, depression, psychoticism, and obsessive-compulsive symptoms eliminated the AFF-RELs vigilance effects, suggesting that working memory deficits in SCZ-RELs were the most robust cognitive impairments [85]. 

A nationwide observational cohort study that included 522 children 7 years of age with no, one, or two parents with a diagnosis of SCZ or BD demonstrated that children with a familial high risk of SCZ presented worse sustained attention compared with control children and children with a familial high risk of BD. Differently, children with a familial high risk of BD displayed similar abilities of sustained attention and interference control as control children, except in terms of a lower accuracy [86]. A similar pattern of cognitive alterations has been reported by a study based on the Swedish Twin Registry that examined cognitive functions in SCZ-RELs and BD-RELs. Here, the authors showed that bipolar co-twins showed superior performance compared with HCs in verbal learning and fluency, while BD showed performance decrements in all neurocognitive domains. These findings did not change when the total score of the Young Mania Rating Scale was included in the model, indicating that the effects were not attributable to clinical symptoms. In contrast, SCZ co-twins exhibited a pattern of attenuated neurocognitive impairments relative to their affected twins [87]. A meta-analysis of cognitive functions in 1341 first-degree SCZ-RELs, 939 BD-RELs, and 1427 HCs demonstrated that SCZ-RELs had cognitive deficits in all domains, while BD-RELs underperformed HCs in processing speed, verbal fluency, and speed-based executive function. A direct comparison highlighted that SCZ-RELs underperformed BD-RELs in general intellectual ability, working memory, verbal memory, planning, processing speed, and fluency [88].

### 5.3. Social Cognition

A large study on 1093 patients with non-affective psychosis, 1044 unaffected siblings, 911 parents, and 587 HCs reported that parents showed alterations in social cognition, in addition to poorer verbal learning, processing speed, reasoning, problem-solving, acquired knowledge, and working memory. Interestingly, the prevalence of clinically relevant cognitive impairment in PSY-RELs ranged from 50% to 10% [89].

## 6. Neuroimaging Findings in SCZ-RELs

### 6.1. Structural Sudies

A large body of evidence suggests that SCZ-RELs present volumetric and cortical alterations that are similar to the structural abnormalities observed in SCZ but are less pronounced. In particular, a recent volume-based meta-analysis of neuroimaging alterations in 6274 SCZ-RELs and 10,789 HCs reported a reduction in total cortical gray matter volume (GMV) and intracranial volume (ICV), in addition to a decrease in thalamic and hippocampal volume in SCZ-RELs [90]. Similarly, another meta-analysis comparing first-degree SCZ-RELs to HCs observed a decrease in GMV and hippocampal volume [91]. The reduction in thalamic volume was also highlighted by the ENIGMA Relatives Working Group [92]. Furthermore, they observed that, after correction for ICV, SCZ-RELs presented smaller total brain, GMV, cerebral white matter, cerebellar gray and white matter, and thalamic volumes [92]. In another article by the same research group, SCZ-RELs had a thinner cortex in most cortical regions compared to HCs, while the same comparison for cortical SA was not statistically significant [75].

Voxel-based morphometry (VBM) studies showed a reduced grey matter volume in SCZ-RELs in the anterior cingulate cortex (ACC), left amygdala and right insula [93], left middle temporal gyrus (MTG), right cerebellum, and right insula [94], right superior frontal gyrus (SFG), left insula, and left thalamus [95]. Differently, Zhang et al. (2020) found an increase in left thalamic volume [94]. Notably, this inconsistency might be due to significant methodological heterogeneities in the studies. Moreover, an increased volume was found in the left medial frontal gyrus [95]. In contrast, a meta-analysis of VBM studies exploring gray matter density in 885 first-degree SCZ-RELs and 775 HCs found no differences between the two groups [96].

In addition to volumetric changes, cortical alterations have also been reported in SCZ-RELs. Particularly, the ENIGMA Relatives Working Group reported a reduction in mean cortical thickness (CT) in SCZ-RELs compared to HCs [92]. Additionally, a study conducted on 200 SCZ-RELs (aged 12–85 years) and 276 unrelated controls suggested early neurodevelopmental effects of the SCZ genetic risk for frontal and insular SA, late neurodevelopmental effects for overall cortical SA, and frontal, parietal, and occipital SA, and possible neurodegenerative effects for temporal CT and parietal SA [97]. Interestingly, a reduction in frontal and temporal SA in SCZ-RELs was correlated with subjective cognitive dysfunction and predisposition to hallucinations [98].

The results of twin studies align with evidence from studies conducted on first- and second-degree SCZ-RELs. In an investigation carried out in 26 twin pairs discordant for SCZ and 83 healthy twin pairs, a higher genetic risk for SCZ was associated with reduced white matter volume, a thinner right orbitofrontal cortex (OFC), a thinner bilateral parahippocampal cortex, and thicker temporoparietal and left superior motor cortices [99]. Interestingly, a longitudinal investigation showed a significant decrease over time in the total brain, frontal, and temporal volumes in patients with SCZ and their unaffected co-twins compared to control twins [100]. Differently, a region-of-interest study that included 21 MZ twin pairs concordant for SCZ, 17 MZ SCZ twins, and 18 MZ non-schizophrenic twins drawn from 19 pairs discordant for SCZ and 26 MZ control twin pairs reported that concordant but not discordant twins with SCZ had significantly lower volumes of the middle frontal cortex and OFC than control twins [101].

### 6.2. Functional Studies

Task-related brain activation and functional connectivity in SCZ-RELs seem to present a pattern of alterations that lie between SCZ and HC. In this section, we discuss the results of investigations using cognitive, emotional, and reward tasks and functional connectivity studies.

#### 6.2.1. Cognitive Tasks

During cognitive tasks, overactivation in SCZ-RELs compared to HCs has been reported in numerous areas, particularly in the frontal, parietal, and temporal lobes. Our research group showed increased activation in the right dorsolateral prefrontal cortex (DLPFC) [90], which was also displayed by other meta-analyses and systematic reviews [102,103]. In addition, the right frontal gyrus [96], the prefrontal cortex (PFC) [102,103,104,105], and the bilateral inferior frontal gyrus (IFG) [102,103,104] were overactivated by cognitive tasks. In the temporal lobe, increased activations were described in the right superior temporal gyrus (STG) [95,106], the right middle temporal gyrus (MTG) [103,105], and the left inferior temporal gyrus (ITG) [102], while, in the parietal lobe, the areas that appeared to be more commonly involved were the right supramarginal and angular gyrus [103], the right superior parietal lobule (SPL) [105], and the left inferior parietal lobe (IPL) [103,105]. Furthermore, increased activation in SCZ-RELs was also reported in the bilateral caudate [102], right precuneus [104], right ACC [105], and bilateral thalamus [106]. Differently, reduced activation has been reported in widespread areas, including the left frontal operculum cortex, the right supplementary motor area, the left insula, the right precentral gyrus, the right postcentral gyrus, the left occipital cortex [102], the right cingulate, the left insula, and the right MTG [104].

#### 6.2.2. Emotive Tasks

A heterogeneous picture of abnormalities in brain activation during emotive tasks has been shown in SCZ-RELs. A meta-analysis that included 12 studies did not show a difference between SCZ-REL and HCs [90]. Similarly, a recent study that employed frequentist and Bayesian analyses in subjects both at clinical and genetic risk for SCZ concluded that brain activation patterns in response to emotional paradigms are unlikely to represent reliable IPs [107]. Differently, a meta-analysis of four studies reported six significant clusters of overactivation in the left sub-gyral parietal lobe, the right SFG, the left lentiform nucleus, the left parahippocampal gyrus, the left precuneus, and the right MTG, along with hypoactivation in the right precentral gyrus, the right IPL, the left MFG, and the right IFG in SCZ-RELs in comparison to HCs [105]. Lastly, a systematic review that collected evidence from three studies reported abnormalities in the amygdala, ACC, and central opercular activation [102].

#### 6.2.3. Reward Tasks

In our recent meta-analysis, we observed that SCZ-RELs showed reduced activation in the ventral caudate compared to HCs during reward tasks [90]. Interestingly, in SCZ offspring, striatal activation during reward anticipation decreased with age, while it did not change in HC, indicating that reducing striatal activation during adolescence may represent a trait of familial vulnerability for SCZ [108]. Differently, an investigation that explored reward processing in twin pairs with at least one twin having a schizophrenia spectrum disorder and control twin pairs from the Danish registries showed that, compared with control twins, unaffected co-twins presented increased activation in the DLPFC during missed target contrast. In co-twins, DLPFC hyperactivation was associated with higher cognitive flexibility [109]. Overall, the limited evidence suggests that fronto-striatal activation abnormalities during rewards paradigms might represent IPs for the disorder.

#### 6.2.4. Functional Connectivity Studies

An increasing body of evidence shows that SCZ-RELs have altered connectivity within and between large-scale brain networks, including executive, default mode, sensory–motor, thalamo-–ortical, and limbic networks [110,111,112]. Additionally, reduced functional connectivity in the ventral and dorsal cortico–striatal circuit has been shown in first-degree SCZ-RELs [113]. Interestingly, abnormalities in brain functional connectivity have also been observed during childhood, suggesting that these alterations are not a result of disrupted late neurodevelopmental processes but appear early in development [114].

Overall, evidence from functional MRI investigations shows a consistent pattern of functional alterations in cortico–striatal–thalamic networks, both in response to specific tasks and during rest, suggesting that cortico–striatal–thalamic network abnormalities might be considered an IP for SCZ.

## 7. Neuroimaging Findings in BD-RELs

### 7.1. Structural Studies

Similar to SCZ-RELs, BD-RELs also show numerous structural alterations in areas that are commonly associated with the disorder. A meta-analysis of structural studies that included 2195 BD-RELs and 3169 HCs showed an increase in GMV in the right IFG and in the left STG and reduced GMV in the right lingual gyrus in BD-RELs compared to HCs. When the studies were clustered according to the age of the participants, adult BD-RELs presented an increased volume in the right IFG, in the left STG, and in the left cerebellum, while child BD-RELs had an increased volume in the left parahippocampal gyrus, along with a decrease in volume in the right cerebellum, in the right SFG, and in the MFG compared to HCs [115]. A meta-analysis of morphometric alterations in BD-RELs revealed decreased GMV in the left temporoparietal junction, the right medium cingulate cortex, the right PFC, and the thalamus in BD-RELs, along with an increased GMV in the right IFG/ventrolateral prefrontal cortex (VLPFC). Notably, increased volume in right VLPFC was present also in adult BD-RELs, BD type I-RELs, and BD-RELs with no psychiatric comorbidities [90]. Consistently with these findings, in a meta-analysis of VBM studies conducted on 2292 relatives of patients with SCZ, BD, and depression and 2052 HCs, BD-RELs exhibited increased GMV in the right IFG, left supramarginal gyrus, and right gyrus rectus, but decreased GMV in the right cerebellum and the right SFG in comparison to HCs [94]. Notably, previous evidence showed a decrease in volume in the supramarginal gyrus in patients with BD [116], indicating a complex relationship between genetic risk and structural changes in this brain region.

The ENIGMA-Relatives Study of Schizophrenia and Bipolar Disorder showed that first-degree BD-RELs had significantly larger ICV compared to HCs. Additionally, first-degree BD-RELs exhibited significantly larger SA, total brain, cortical gray matter, cerebral white matter, cerebellar gray matter, thalamus, and accumbens volumes compared to HCs [92]. The same research group observed that, after correction for mean CT, first-degree BD-REL showed a significantly increased CT in the right caudal MFG. In addition, the study revealed larger cortical SA in many cortical areas, including the left transverse temporal, left parahippocampal, right STG, right supramarginal gyrus, and right transverse temporal gyrus [75]. These results are in line with evidence from smaller studies [117,118]. Interestingly, it has been demonstrated that BD-RELs show accelerated cortical thinning and volume reduction in the right frontal regions, including the IFG, lateral OFC, frontal pole, and rostral MFG compared to HCs, which is similar to what has been observed in BD [118]. Lastly, a recent systematic review by our research group showed volumetric and surface differences in the IFG, but no alterations in GI [119].

Overall, this evidence seems to support the prefrontal and the temporal lobe, and, in particular, the IFG, SFG, MFG, and STG as key regions associated with genetic risk for BD.

### 7.2. Functional Studies

In BD-RELs, a consistent pattern of cortico–limbic alterations was observed in var-ious meta-analyses and systematic reviews.

#### 7.2.1. Cognitive Tasks

Our research group demonstrated that, compared to HCs, BD-RELs exhibited an overactivation of the right caudate, right IFG, dorsal ACC, and left MTG/STG [115], as well as in the right posterior cingulate cortex (PCC) and the right anterior PFC, in association with an underactivation of the bilateral superior parietal lobule and the left postcentral gyrus [90] during cognitive paradigms that included attention, memory, and executive functions. In addition, a systematic review reported that studies on BD-RELs consistently found hyper-activity relative to HCs, mostly involving the prefronto–striatal circuit during cognitive control tasks, regardless of paradigm design and task contrast [102]. Differently, during working memory tasks, BD-RELs showed a pattern of increased and decreased activation in the VLPFC and frontopolar cortex [102]. Notably, the potential role of alerted OFC activity during working memory tasks was also suggested by twin studies [120].

#### 7.2.2. Emotive Tasks

Meta-analytic evidence has consistently shown an increased activation of BD-RELs in the limbic lobe, and, in particular, in the right parahippocampal gyrus/uncus [90,115], and the right amygdala [115]. Interestingly, studies employing similar emotion processing tasks found that BD-RELs had amygdala hyperactivation when viewing faces and rating them as fearful versus passive viewing, during viewing emotional faces versus shapes, and during successful versus unsuccessful encoding of emotional faces [102]. Additionally, during emotive tasks, BD-RELs also showed altered activation in IFG/MFG, suggesting an impairment in the fronto–limbic circuit [102]. In twins, a hypoactivation of the posterior dorsal ACC and the left frontal areas during reappraisal of unpleasant pictures was observed in unaffected MZ twins with a co-twin history of unipolar or BD compared to HCs [121]. Therefore, increased activity in the limbic lobe and hypoactivation in the frontal areas during emotion processing, which is also a common finding in BD [122], could be considered an IP for the disorder.

#### 7.2.3. Reward Tasks

Hyperactivation of the left lateral OFC [102], anterior PFC, and the OFC [115] and hypo-activation in the right pregenual cingulate cortex [102] have been reported in BD-RELs during reward-processing tasks. Overactivation was also described in the left SFG, MFG, and left insula, regardless of the task performed during MRI [123].

#### 7.2.4. Functional Connectivity Studies

A recent review conducted by our research group identified 10 studies that explored resting state functional connectivity and spontaneous brain activity in BD-RELs. We reported that the most frequently affected region was the amygdala. In particular, numerous studies found altered connectivity between the amygdala and DLPFC and the left ITG. Interestingly, studies on RELs of psychotic BD showed more widespread alterations, involving the fronto–occipital network, the fronto–thalamic–striatal network, and the DMN [124].

## 8. Neuroimaging Findings in PSY-RELs

### 8.1. Structural Studies

Evidence on structural brain abnormalities in PSY-RELs is still scarce. A meta-analysis of brain structural correlates of familial risk for SCZ, BD, and major depressive disorder showed that, when RELs of patients with SCZ, BD, and major depressive disorder were considered as a whole, GMV reduction in the right cerebellum appeared as a common brain structural abnormality present in all RELs. Importantly, in this study depressed patients did not necessarily present psychotic symptoms [94]. A recent MRI investigation showed that siblings of FEP presented significantly smaller left lateral, right lateral, and third ventricle volumes compared to FEP [125]. With regards to cortical measures, in a sample of first-degree relatives of SCZ, SZA, and BD patients with cluster A personality disorders, nearly significant hypogyria was seen in the PCC and in the bilateral caudal ACC compared with HCs [126]. Interestingly, a longitudinal study carried out on offspring of SCZ and BD demonstrated that PSY-RELs who developed psychotic spectrum symptoms over the course of follow-up showed a greater time-related reduction in mean CT than those who did not and HCs. By subgroups, this effect was present in both offspring of SCZ and BD in the occipital cortex [127].

### 8.2. Functional Studies

The literature exploring functional brain abnormalities in PSY-RELs is limited. A small study conducted on children at familiar high risk for both affective and non-affective psychosis demonstrated that, during a working memory task, child PSY-RELs exhibited hypoactivation in the 2-back condition in a cluster encompassing bilateral precuneus and cuneus and right PCC compared to HCs [83]. Neural response to an interactive task was explored in 50 healthy siblings of patients with psychosis recruited from the Dutch Genetic Risk and Outcome in Psychosis study and 33 HCs. The authors observed that the siblings showed hypoactivation in the right striatum during investments and the left insula during repayments [128]. Lastly, a recent study reported that patients with schizophrenia-spectrum disorder or psychotic BD and their first-degree RELs presented similar patterns of dysconnectivity involving the thalamo–prefrontal–cerebellar and thalamo–insular regions. Interestingly, the authors observed that these alterations were associated with clinical symptoms and cognitive deficits both in patients and in their relatives [129].

With regards to studies directly comparing SCZ-RELs and BD-RELs, our meta-analysis demonstrated that BD-RELs had higher activation in the right VLPFC and right parahippocampal gyrus compared to SCZ-RELs. Additionally, BD-RELs had a higher probability of functional and structural convergent alterations in the right VLPFC compared to SCZ-RELs, while SCZ-RELs had a greater likelihood of convergent functional MRI and VBM alterations in the right DLPFC compared to BD-RELs [90].

A functional MRI investigation during reward-processing in patients (SCZ: *n* = 46; BD: *n* = 45) and unaffected first-degree RELs (SCZ: *n* = 46; BD: *n* = 50) and 396 HCs explored disorder-specific differences in functional ventral striatum–hippocampus coupling. The authors reported that the functional ventral striatum–hippocampus coupling was reduced in SCZ and BD, as well as in first-degree SCZ-RELs, suggesting that this could represent an IP for SCZ. No alterations in BD-RELs were reported [130].

Another large investigation that included 220 SCZ, 147 SAD, 180 psychotic BD, 150 first-degree SCZ-REL, 126 SAD-RELs, 134 BD-RELs, and 242 HCs observed that the association between functional alterations in a prefrontal–striatal–thalamic–cerebellar network and structural abnormalities in the DMN was common across psychotic diagnoses, but it was not present in the RELs, indicating that this might represent a marker of the disorder rather than an IP [131]. Lastly, a resting-state study that explored spontaneous brain activity in SCZ and psychotic BD, their RELs, and HCs found no alterations in SCZ-RELs and psychotic BD-RELs compared to HCs, but observed that psychotic BD-RELs had increased network connectivity relative to HCs between the left precentral/postcentral gyrus and bilateral caudate [132].

## 9. Limitations

The present narrative review presents some limitations. First, we were unable to include quantitative analyses, systematic methodology, the risk of bias, or other quality assessments of the included studies that could be performed with meta-analytic approaches, which were beyond the scope of this paper. Secondly, although we used comprehensive search strategies to summarize and synthesize the existing available knowledge, we may not have identified all of the relevant literature and may have been subjected to selection bias. Nonetheless, we have used as sources the results from all the meta-analyses that were previously published on these topics. Accordingly, our review can provide an overview on consistent alterations from two different and complementary research fields, which can contribute to hypothesis generation for future studies.

## 10. Conclusions

In conclusion, individuals at genetic risk for SCZ and BD present a pattern of neuropsychological and neuroimaging alterations that lie between patients and HCs. In particular, deficits in cognitive functions, including intelligence, memory, attention, executive functions, and social cognition, can be considered IPs for SCZ and could potentially help identify RELs at particular risk of developing the disorder. Although the picture for cognitive alterations in BD and BD-RELs is less clear compared to SCZ, the available literature seems to suggest that BD-RELs present some specific deficits in executive functioning, including adaptable thinking, planning, self-monitoring, self-control, and working memory. Additionally, abnormal cognitive flexibility could be associated with the risk of psychotic BD, while impairments in attention, verbal memory, and executive functions could represent predictive factors for the development of mood episodes. Studies that directly compared cognitive functioning in the two REL groups demonstrated that SCZ-RELs underperformed BD-RELs in general intellectual ability, working memory, verbal memory, planning, processing speed, and fluency, suggesting that the genetic risk for SCZ is linked to a more substantial pattern of cognitive deficits compared to the genetic risk for BD. Furthermore, both SCZ-RELs and BD-RELs present structural and functional alterations that are similar to the abnormalities observed in patients, though they are less pronounced. In particular, SCZ risk appears to be associated with alterations in the cortico–striatal–thalamic network, which may result in altered cognitive processing, goal-directed behavior, and evaluation of salient information. Differently, BD risk is associated with changes in areas that play a crucial role in emotional regulation, including the prefrontal, temporal, thalamic, and limbic regions. The direct comparison between neuroimaging findings in SCZ-RELs and BD-RELs suggests that the risk of both disorders is linked to alterations in the cortico–thalamic circuits, with the involvement of different prefrontocortical and temporal regions associated with differential clinical expression in SCZ and BD. Similar neuropsychological and neuroimaging abnormalities in SCZ-RELs and BD-RELs may be the phenotypic expression of the shared genetic mechanisms underlying both disorders, while the specificities in neuropsychological and neuroimaging profiles may be associated with the differential symptom expression in the two disorders.

Overall, IPs hold great promise in prevention research because they can be used to identify those individuals at genetic risk who are at ultra-high risk of developing psychiatric disorders. In particular, longitudinal assessment of IPs could enable clinicians and researchers to monitor changes in cognitive functioning and neuroimaging measures over time, thus identifying markers with sufficient sensitivity and specificity that can predict outcomes at the individual level. To broaden our knowledge, genetic, neuroimaging, cognitive, and clinical data need to be combined in large samples to identify multiple small effects that vary between individuals, ultimately enabling us to understand and predict the course of SCZ and BD and facilitating early intervention strategies. 

## Figures and Tables

**Table 1:** Main neuropsychological and neuroimaging findings in SCZ-RELs and BD-RELs.

	SCZ-RELs	BD-RELs
**Neuropsychological findings**		
Intelligence	- Lower IQ compared to the general population - Superiority of verbal skills over spatial skills	- Lower IQ with preserved educational performance
Memory, attention, and executive functions	- Deficits in working memory and short-term memory - Deficits in cognitive flexibility - Deficits in executive functions	- Deficits in executive functioning, including adaptable thinking, planning, self-monitoring, and self-control - Deficits in memory and cognitive flexibility - Deficits in executive functions - Deficits in working memory
Social cognition	- Impaired mentalization - Substandard use of the theory of mind - Reduced ability in emotional processing and perception - Reduced emotional intelligence - Reduced ability to detect sarcasm	- Impaired mentalization
**Neuroimaging findings**		
Structural studies	- Reduced total GMV - Reduced total brain volume - Reduced ICV - Diffused cerebral cortex thinning - Reduced cerebral white matter - Reduced volume in the thalamus and hippocampus - Reduced cerebellar white and gray matter - Reduced volume in the ACC, left amygdala, bilateral insula, left thalamus, left MTG, and right SFG - Increased volume in the left thalamus and left MFG - Reduced CT in the right OFC and the parahippocampal cortex - Increased CT in the temporoparietal cortex and left superior motor cortex - Decreased total brain and frontal and temporal volume over time	- Increased cerebral surface area - Increased cortical gray matter, cerebral white matter, and cerebellar gray matter - Increased ICV - Increased volume in right IFG, right VLPFC, left STG, left supramarginal gyrus, and right gyrus rectus - Reduced volume in the bilateral thalamus, right SFG, right PFC, right MCC, left temporoparietal junction, right lingual gyrus, and right cerebellum - Increased volume in the thalamus and nucleus accumbens - Reduced CT in the right caudal MFG - Reduced CT and volume in the IFG, lateral OFC, frontal pole, and rostral MFG over time
Functional studies	**Cognitive tasks** - Overactivation in the right frontal gyrus, bilateral frontopolar PFC, bilateral IFG, right DLPFC, right STG, right MTG, left ITG, right supramarginal and angular gyrus, right SPL, left IPL, bilateral caudate, right precuneus, right ACC, and the bilateral thalamus - Underactivation in the left frontal operculum, right supplementary motor area, right precentral gyrus, right postcentral gyrus, right MTG, left occipital cortex, right cingulate, and left insula	**Cognitive tasks** - Overactivation in the right anterior PFC, right IFG, left MTG/STG, dorsal ACC, right PCC, and right caudate - Underactivation in bilateral SPL and left postcentral gyrus
	**Emotive tasks** - Overactivation in the right SFG, left sub-gyral parietal lobe, right MTG, left precuneus, left lentiform nucleus, and left parahippocampal gyrus - Underactivation in the left MFG, right IFG, right precentral gyrus, and right IPL	**Emotive tasks** - Overactivation in the right parahippocampal gyrus/uncus, and right amygdala - Underactivation in the IFG, MFG, and posterior dorsal ACC
	**Reward tasks** - Overactivation in the DLPFC - Underactivation in the ventral caudate	**Reward tasks** - Overactivation in the left OFC, left SFG, MFG, and left insula - Underactivation in the right pregenual cingulate cortex
	**Functional connectivity** - Increase FC in the executive, DMN, sensory-motor, limbic, and thalamo-cortical network - Decrease FC in the ventral and dorsal cortico–striatal circuit	**Functional connectivity** - Altered FC between the amygdala and DLPFC and the left ITG - Widespread FC alterations, involving the fronto-occipital network, the fronto–thalamic–striatal network, and the DMN

ACC: anterior cingulate cortex, BD-RELs: relatives of individuals with bipolar disorder, CT: cortical thickness, DLPFC: dorsolateral prefrontal cortex, DMN, default mode network, FC: functional connectivity; GMV: gray matter volume, HC: healthy controls, ICV: intracranial volume, IFG: inferior frontal gyrus, IPL: inferior parietal lobule, IQ; intelligence quotient, ITG: inferior temporal gyrus, MCC: middle cingulate cortex, MFG: middle frontal gyrus, MTG: middle temporal gyrus, MZ: monozygotic, OFC: orbitofrontal cortex, PCC: posterior cingulate cortex, PFC: prefrontal cortex, SA: surface area, SCZ-RELs: relatives of individuals with schizophrenia, SFG: superior frontal gyrus, SPL: superior parietal lobule, STG: superior temporal gyrus, VLPFC: ventrolateral prefrontal cortex.

## Data Availability

Not applicable.
